# Supplementary material for: Hierarchical Clustering and Trajectory Analyses Reveal Viremia-Independent B-Cell Perturbations in HIV-2 Infection
Source: Cells. 2022 Oct 6;11(19):3142. doi: 10.3390/cells11193142 (PMC9562922; doi:10.3390/cells11193142)
Supplement: Supplementary file 1 [file cells-11-03142-s001.zip › cells-1922271-supplementary/Johansson Supplementary Material.pdf]

Hierarchical clustering and trajectory analyses reveal viremia-independent B-cell perturbations in HIV-2 infection

Emil Johansson[1*], Priscilla F. Kerkman[2,3], Lydia Scharf[4], Jacob Lindman[5], Zsófia I. Szojka[1], Fredrik Månsson[6], Antonio Biague[7], Patrik Medstrand[6], Hans Norrgren[5], Marcus Buggert[8], Annika C. Karlsson[4], Mattias N.E. Forsell[2,†], Joakim Esbjörnsson[6,9†], Marianne Jansson[1,†] and the SWEGUB CORE Group

1. Department of Laboratory Medicine, Lund University, Lund, Sweden
2. Department of Clinical Microbiology, Umeå University, Umeå, Sweden
3. Department of Medical Microbiology, University Medical Center Utrecht, Utrecht University, Utrecht, The Netherlands
4. Department of Laboratory Medicine, Karolinska Institutet, Stockholm, Sweden
5. Department of Clinical Sciences Lund, Lund University, Lund, Sweden
6. Department of Translational Medicine, Lund University, Lund, Sweden
7. National Laboratory for Public Health, Guinea-Bissau
8. Department of Medicine Huddinge, Karolinska Institutet, Stockholm, Sweden
9. Nuffield Department of Medicine, University of Oxford, Oxford, United Kingdom.

* Correspondence: emil.johansson@med.lu.se

† These authors have contributed equally to this work.

Supplementary Material

Supplementary Tables

Table S1. Description of antibodies used for flow cytometry.

Antigen	Fluorochrome	Clone	Supplier
CD71	BV421	M-A712	BD Biosciences
CD3	V500	SK7	BD Biosciences
CD14	V500	M5E2	BD Biosciences
CD27	BV570	O323	Biolegend
T-bet	BV605	4B10	Biolegend
CD38	BV711	HIT2	BD Biosciences
CD19	BC786	SJ25C1	BD Biosciences
CD95	Pe-CF594	DX2	BD Biosciences
CD20	PE-Cy5	DX2	Biolegend
HLA-DR	BUV395	G46-6	BD Biosciences

Supplementary Figure legends

Figure S1. Gating strategies. (A) Representative gating strategy to identify $CD19^{+}CD20^{+}$ B-cells, where these cells were further characterized based on CD95, T-bet and CD27 expression. **(B)** Representative gating strategy for the identification of CD19+ B-cells for subsequent bioinformatical analysis.

Figure S2. Marker expression and UMAP location of FlowSOM clusters. (A) Expression of each marker on the 16 different FlowSOM clusters. Cluster name and frequency across all samples are indicated to the left of each histogram row. Pooled cells from all samples are displayed as the reference sample. Median expression intensity of this pooled sample is displayed as a black line. **(B)** FlowSOM clusters **2, 6, 8, 9 and 11,** found to distinguish HIV infected from HIV seronegative individuals, projected on UMAP plots faceted by HIV status groups.

Figure S3. Frequency of T-bet^{high} clusters correlate with markers of disease progression and inflammation. (A) Correlation between cluster 8 (top row) and cluster 9 (bottom row) and IgG1 and IgG3 plasma concentration in HIV-1 infected individuals (n=15). **(B)** Correlation between Cluster 8 and IgG1 plasma concentration in HIV-2 infected individuals (n=20). **(C)** Correlation between Cluster 8 frequency and HIV-2 viral load, CD4%, and plasma concentration of IL-12, IL-18, TNF-α, IFN-γ, CXCL9, and CXCL10 among HIV-2 infected individuals (n=15). Statistically significant correlations were determined using the Spearman rank correlation test. Cytokine concentration is reported as normalized protein expression (NPX) levels. Filled circles refer to treatment naïve or sub-optimally ART treated HIV-1, open circles to successfully ART treated HIV-1, filled squares to viremic HIV-2 and open squares to aviremic HIV-2 infected individuals.

Figure S4. HIV-1 and HIV-2 infection induces activation of naïve-like B-cells. Frequency of FlowSOM cluster 3 (left dot plot) and ratio between frequency of FlowSOM cluster 3 and 2 (right dot plot) from treatment naïve or sub-optimally ART treated HIV-1 (Viremic HIV-1, n=8), successfully ART treated HIV-1 (ART HIV-1, n=7), viremic HIV-2 (Viremic HIV-2, n=8), aviremic HIV-2 (Aviremic HIV-2, n=12) infected individuals and HIV seronegative (seronegative, n=25) individuals. Nonparametric Kruskal–Wallis test followed by Dunn's post-hoc test was performed to compare groups. Medians and IQR are depicted in dot plots.

Figure S5. Slingshot-defined lineage faceted by HIV status group. **(A)** Pseudotime lineages projected on UMAP plots faceted by HIV status groups. **(B)** Jitterplots displaying FlowSOM clusters along pseudotime for each HIV status group. Samples were obtained from treatment naïve or sub-optimally ART treated HIV-1 (Viremic HIV-1, n=8), successfully ART treated HIV-1 (ART HIV-1, n=7), viremic HIV-2 (Viremic HIV-2, n=8), aviremic HIV-2 (Aviremic HIV-2, n=12) infected individuals and HIV seronegative (seronegative, n=25) individuals.

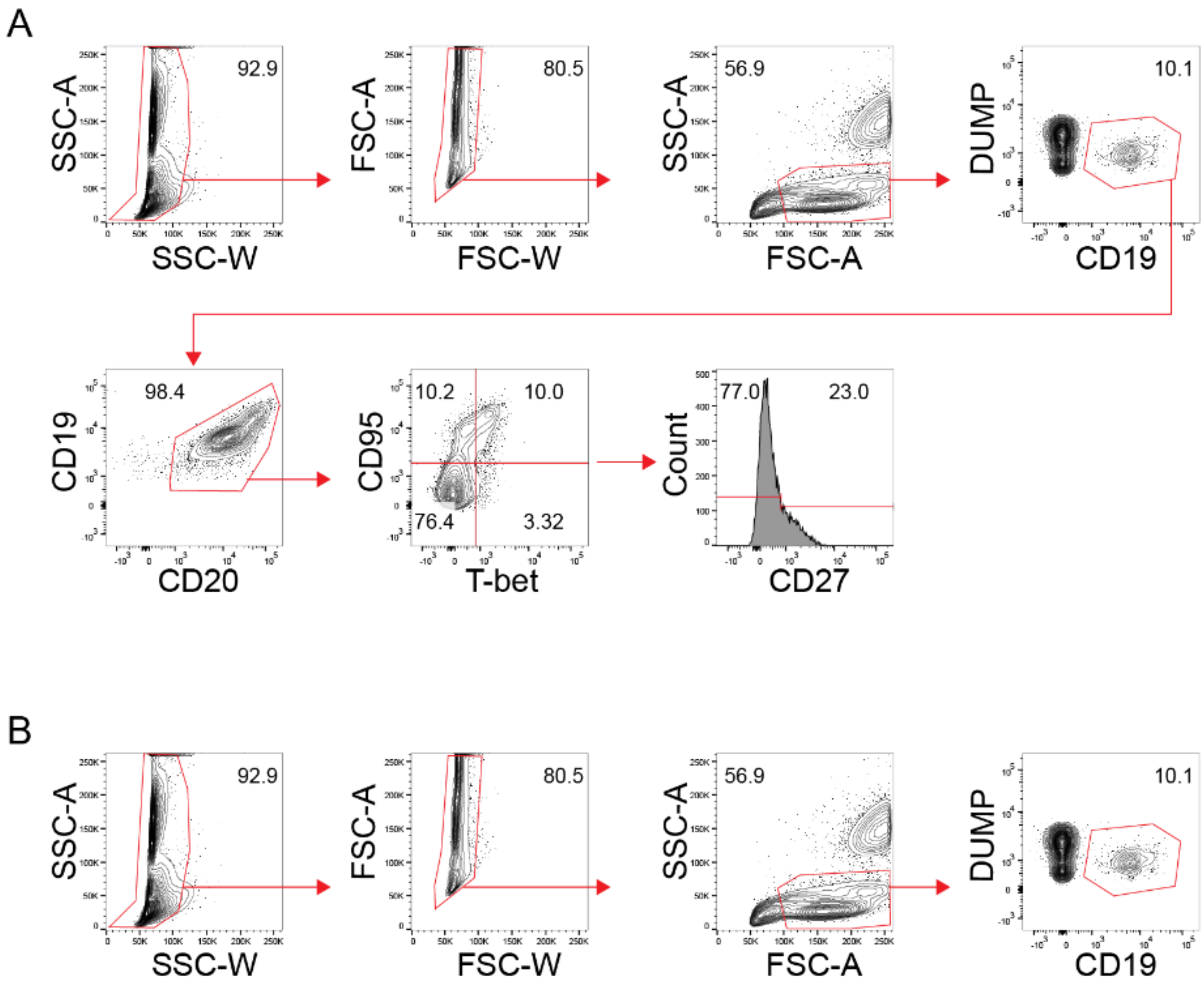

Figure S1. Gating strategies

(A)

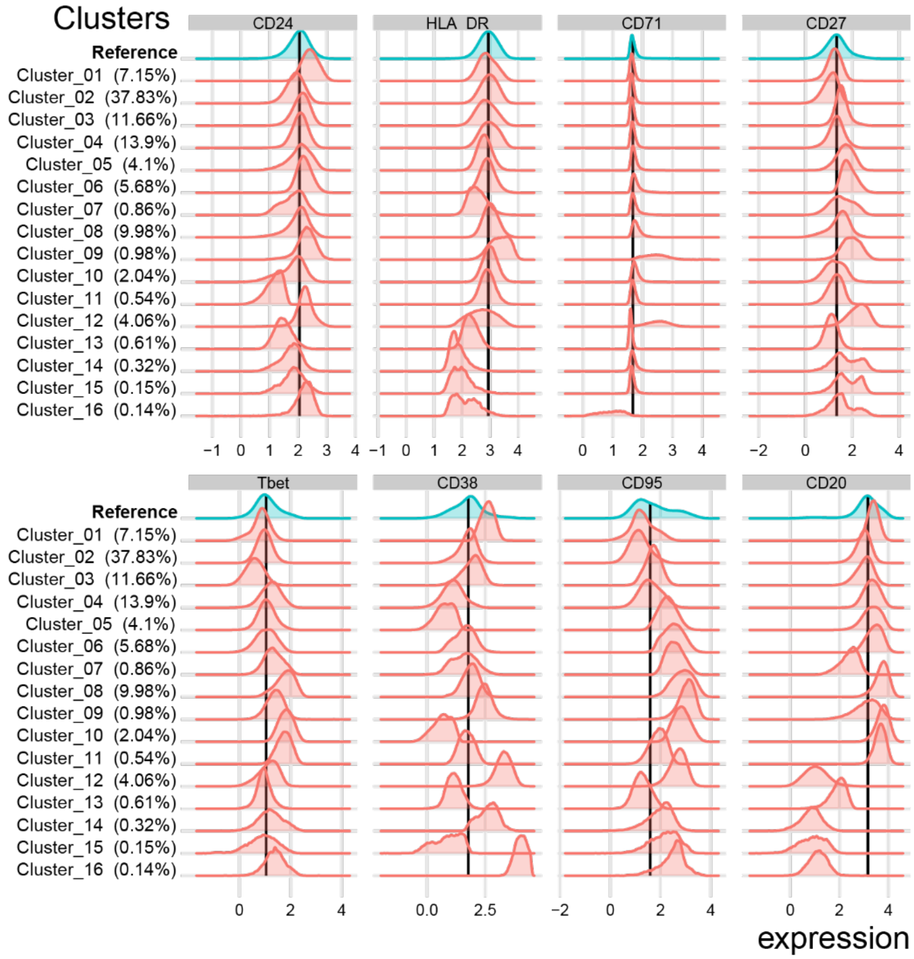
Clusters
CD24
HLA DR
CD71
CD27
Tbet
CD38
CD95
CD20
Reference
Cluster_01 (7.15%)
Cluster_02 (37.83%)
Cluster_03 (11.66%)
Cluster_04 (13.9%)
Cluster_05 (4.1%)
Cluster_06 (5.68%)
Cluster_07 (0.86%)
Cluster_08 (9.98%)
Cluster_09 (0.98%)
Cluster_10 (2.04%)
Cluster_11 (0.54%)
Cluster_12 (4.06%)
Cluster_13 (0.61%)
Cluster_14 (0.32%)
Cluster_15 (0.15%)
Cluster_16 (0.14%)
expression

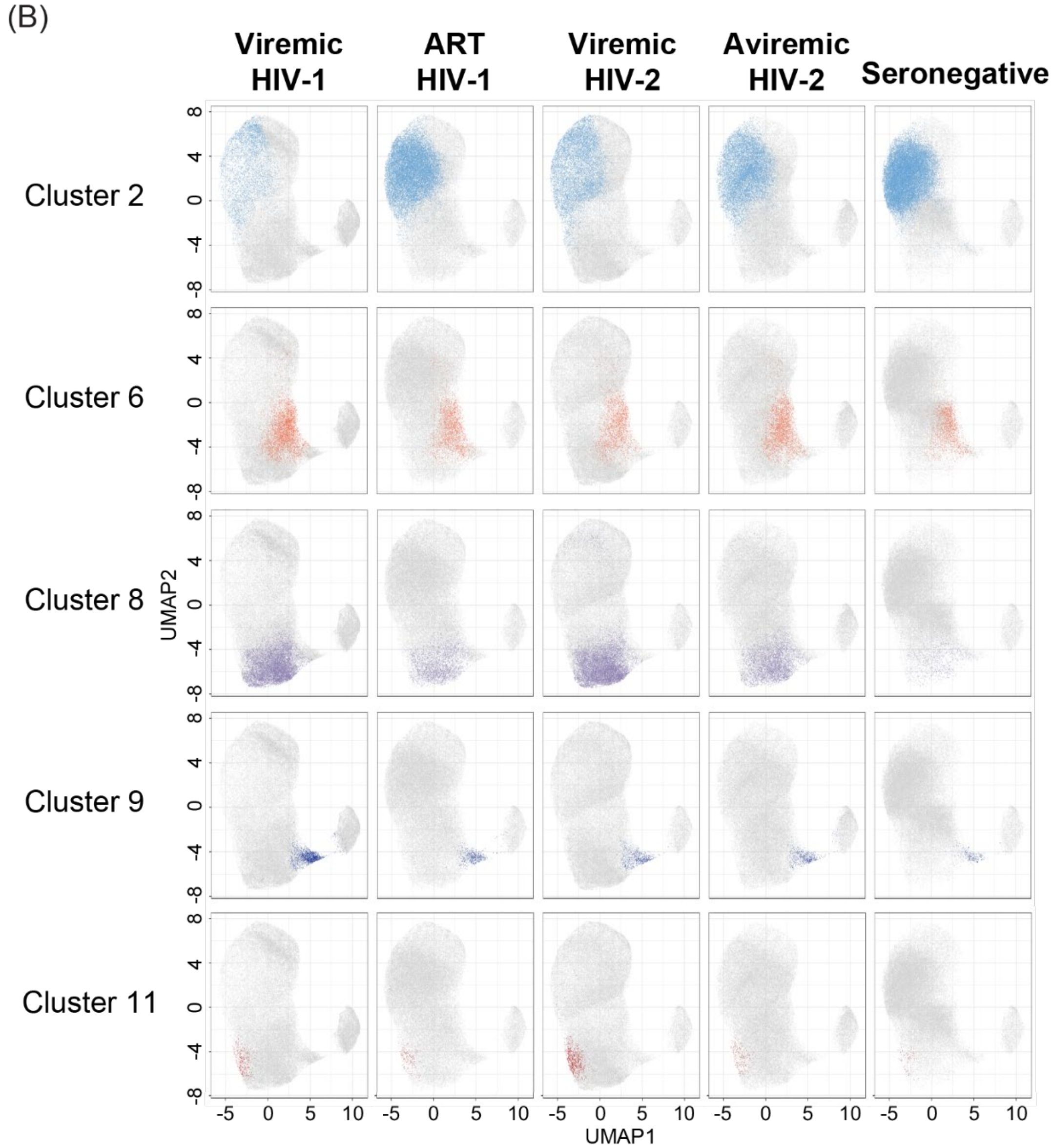

Figure S2. Marker expression on B-cells in FlowSOM clusters.

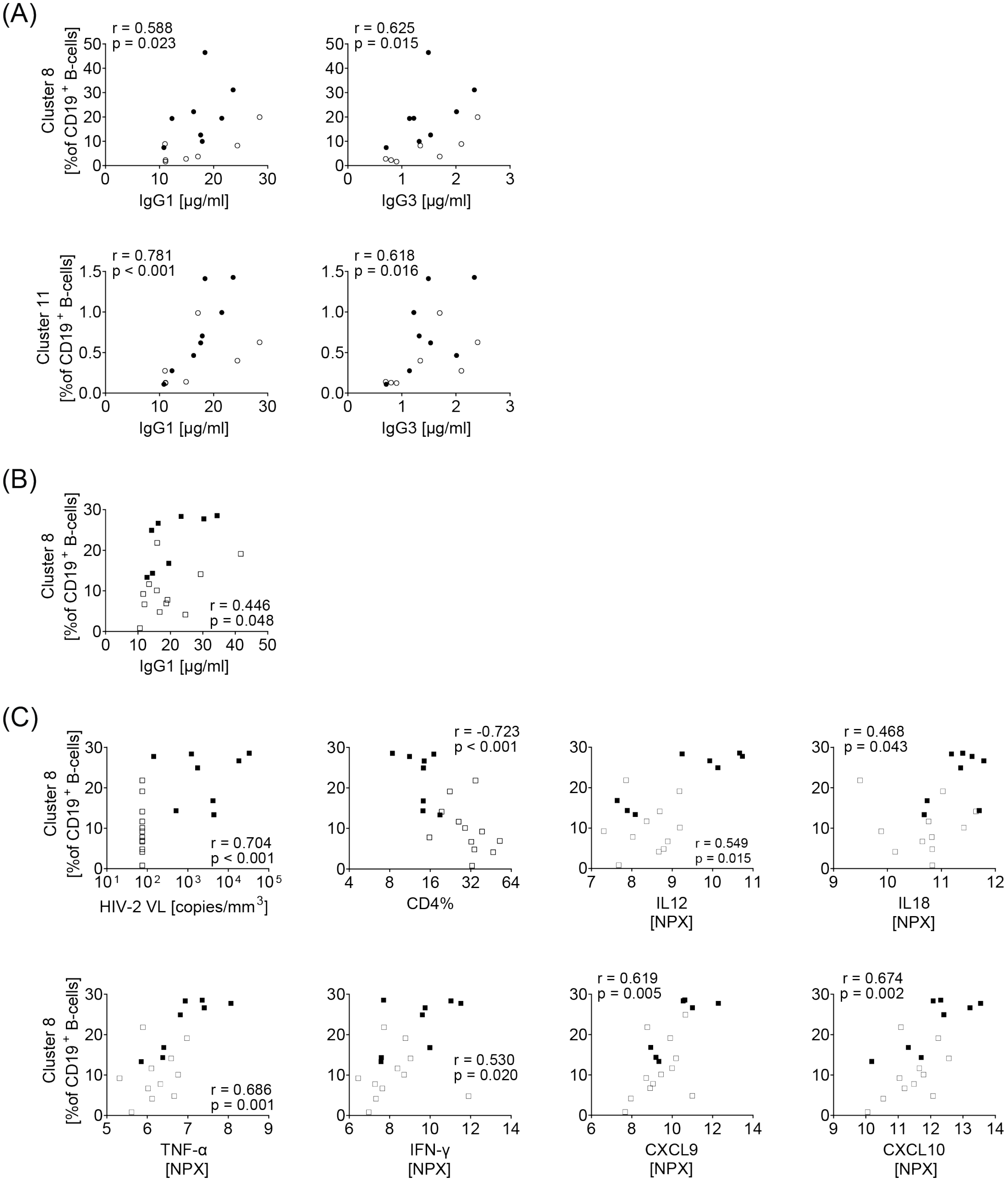

Figure S3. Frequency of T-bet^{high} clusters correlate with markers of disease progression and Th1-associated inflammation.

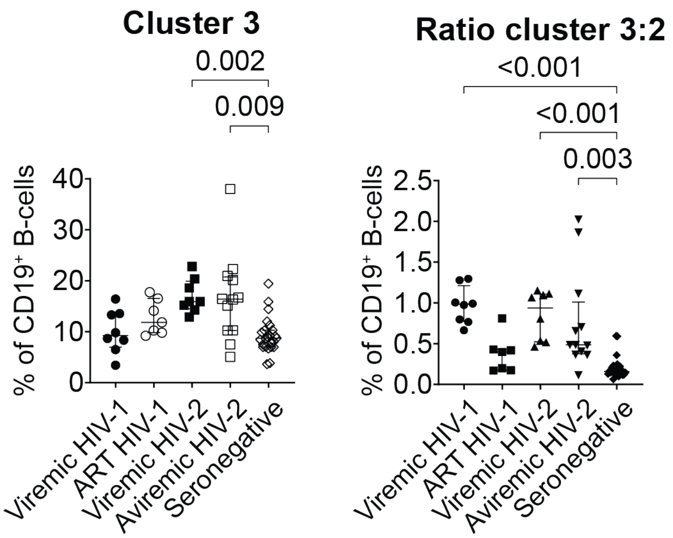

Figure S4. HIV-1 and HIV-2 infection induces activation of naïve-like B-cells.

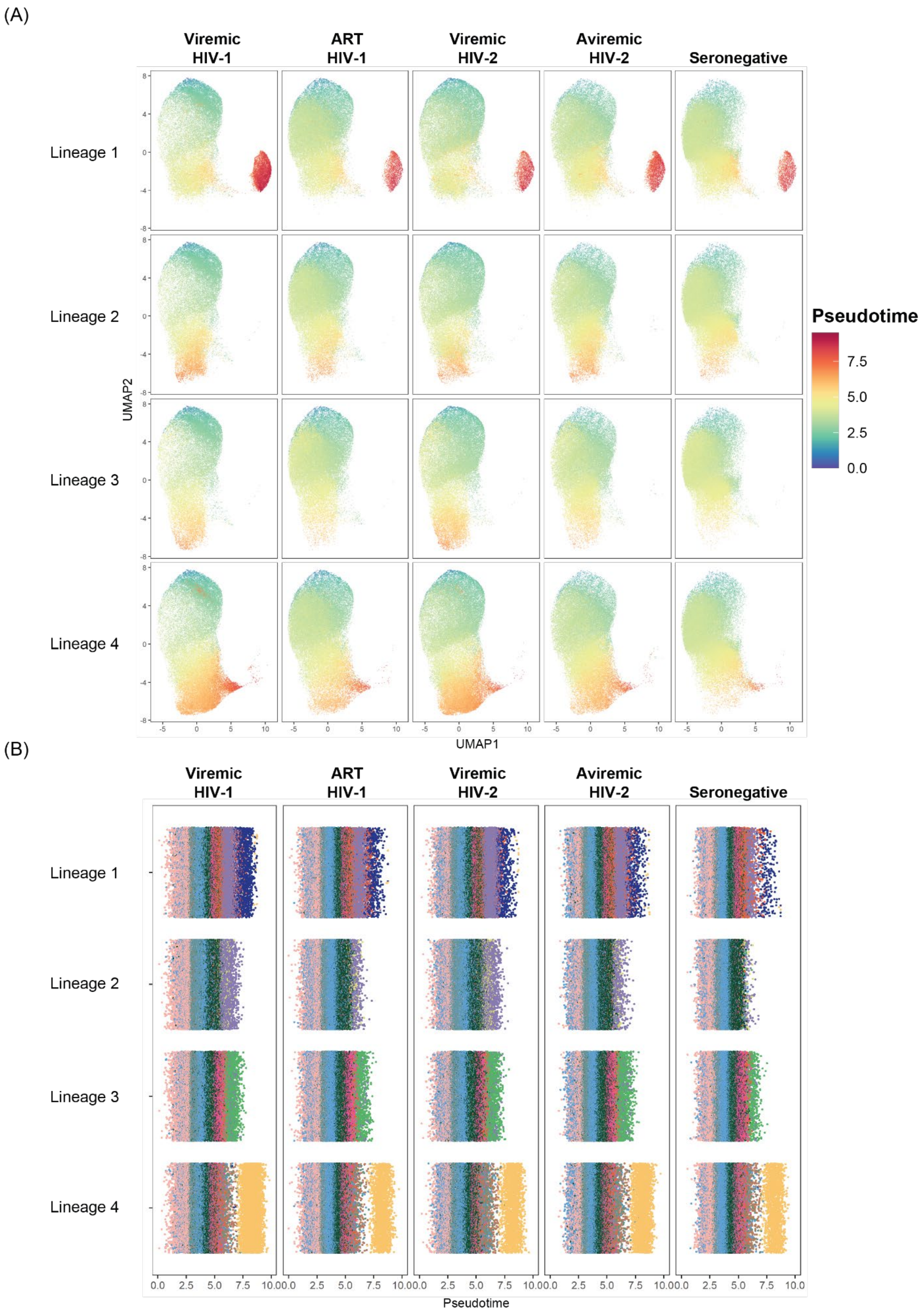

Figure S5 Slingshot-defined lineage faceted by HIV status group.
