# Supplementary figures and images for: Hierarchical Clustering and Trajectory Analyses Reveal Viremia-Independent B-Cell Perturbations in HIV-2 Infection
Source: Cells. 2022 Oct 6;11(19):3142. doi: 10.3390/cells11193142 (PMC9562922; doi:10.3390/cells11193142)

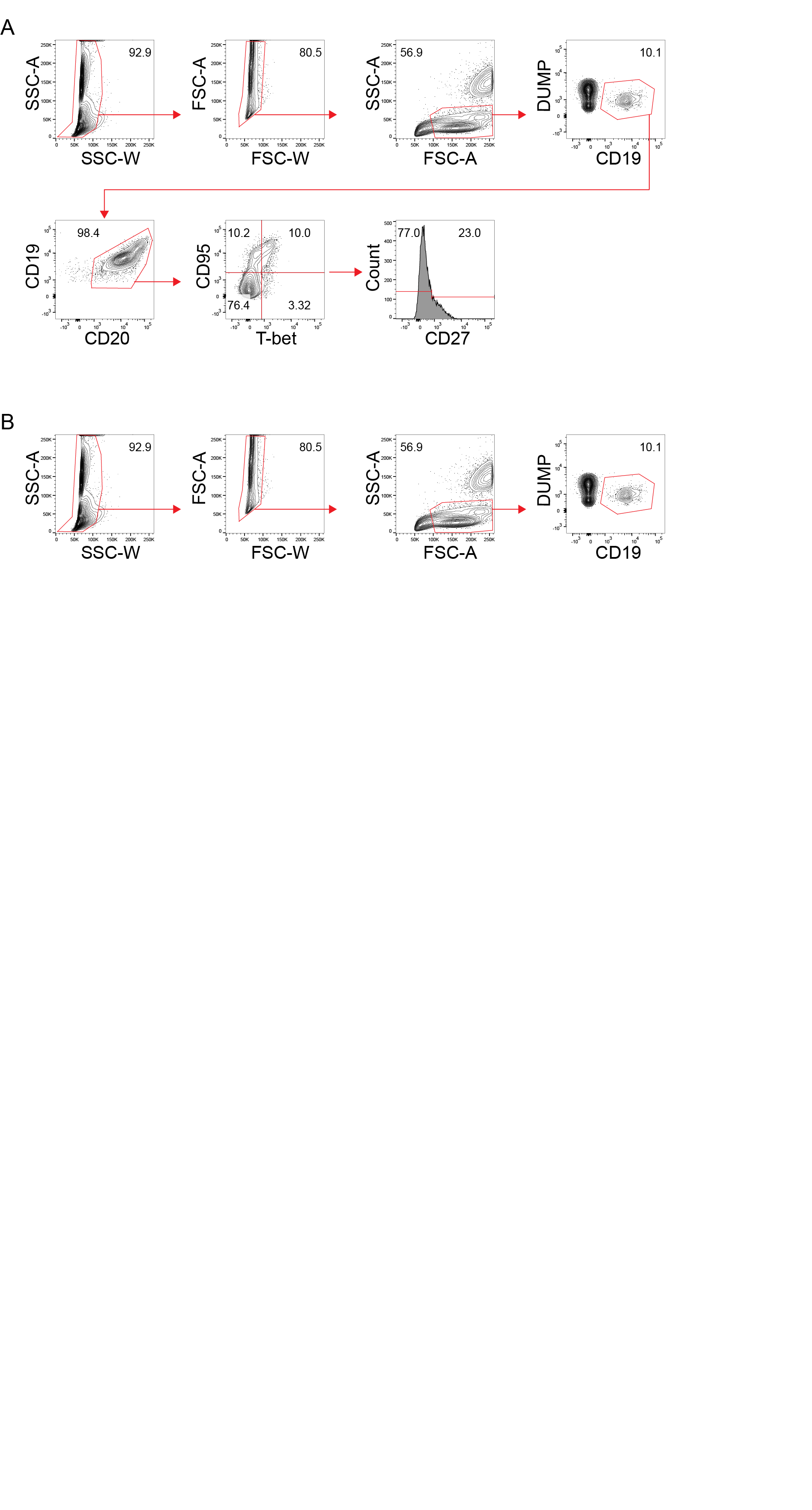

Supplement: Supplementary file 1 [file cells-11-03142-s001.zip › cells-1922271-supplementary/Figure S1.png]

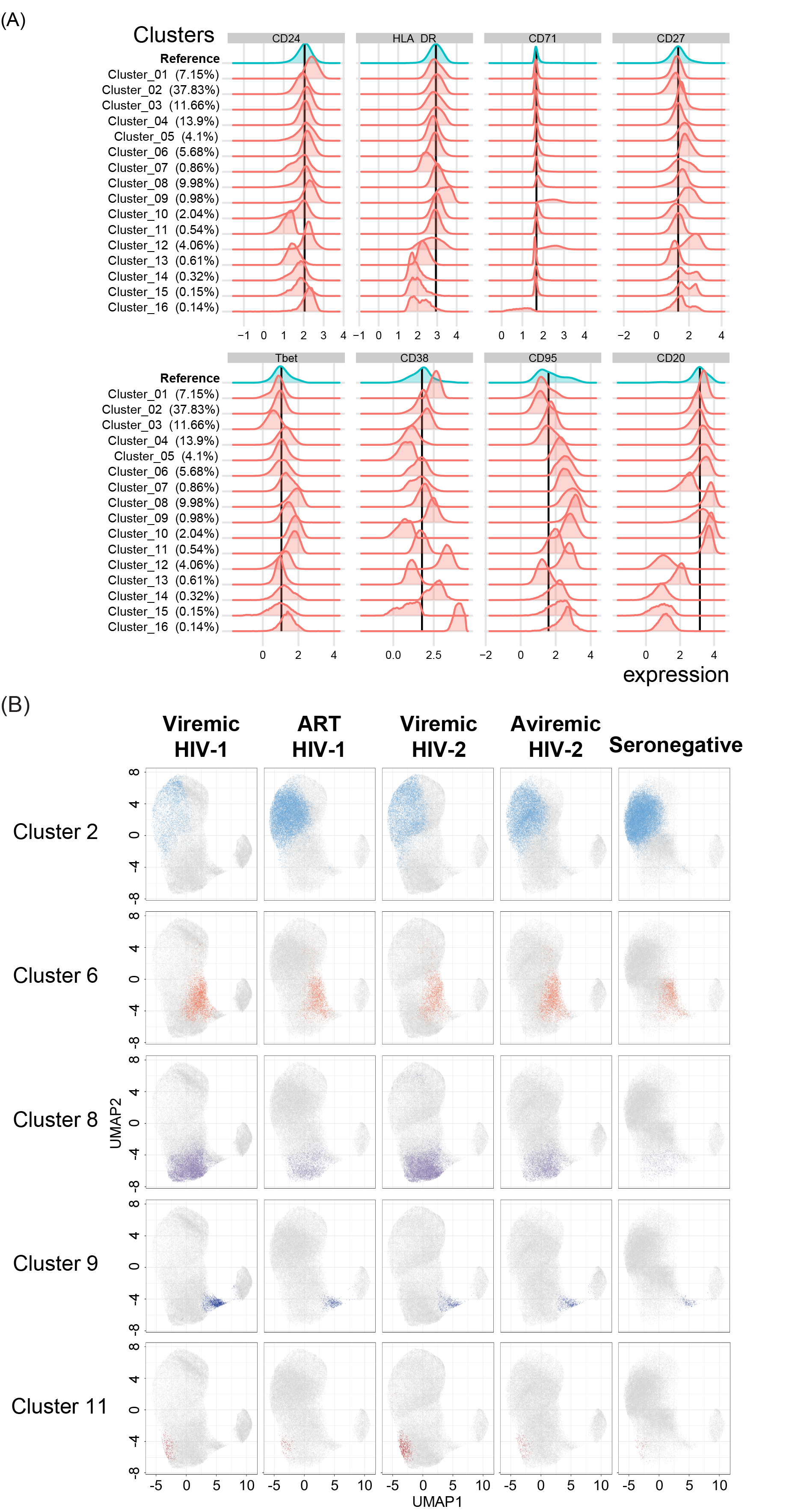

Supplement: Supplementary file 1 [file cells-11-03142-s001.zip › cells-1922271-supplementary/Figure S2.png]

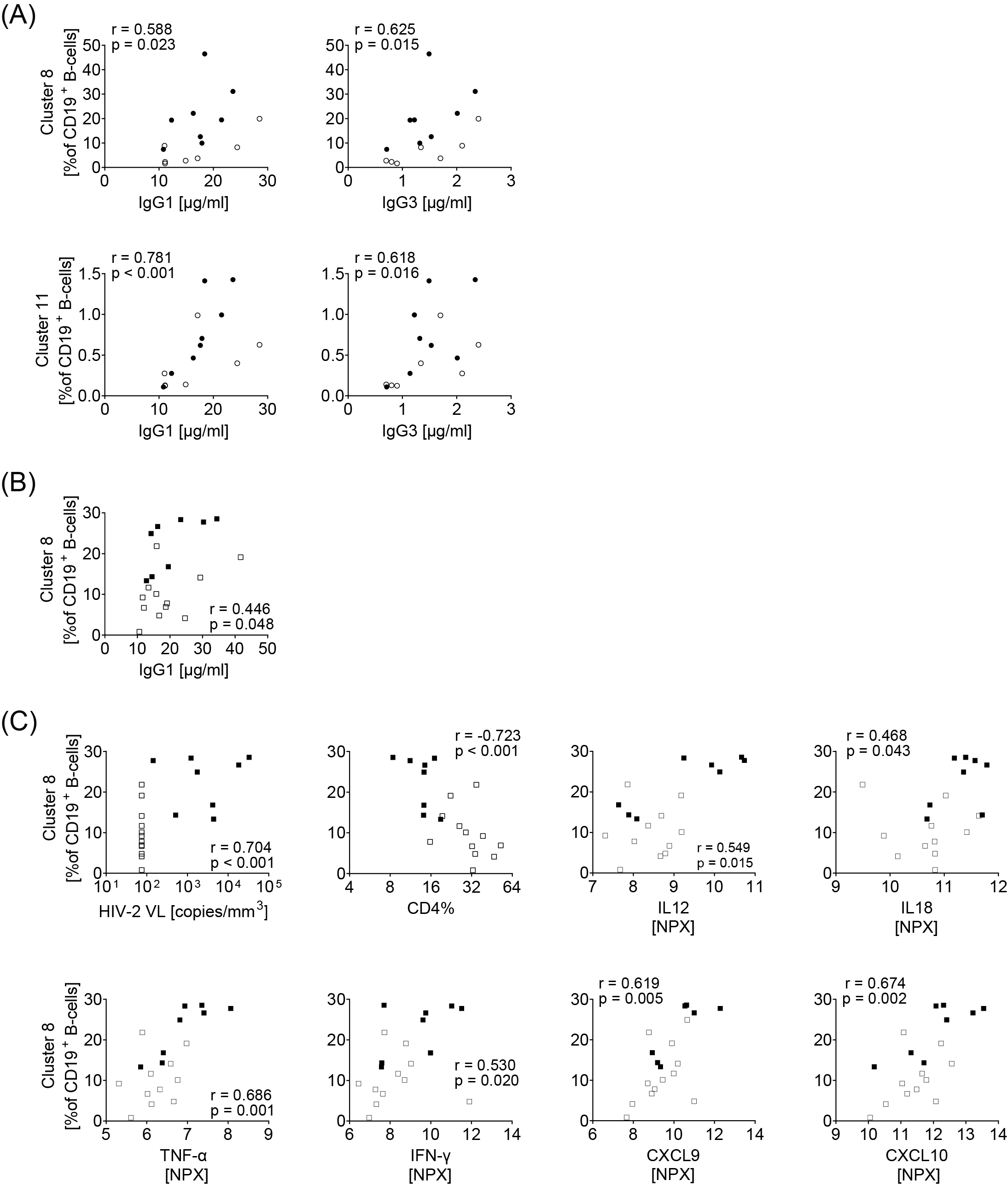

Supplement: Supplementary file 1 [file cells-11-03142-s001.zip › cells-1922271-supplementary/Figure S3.png]

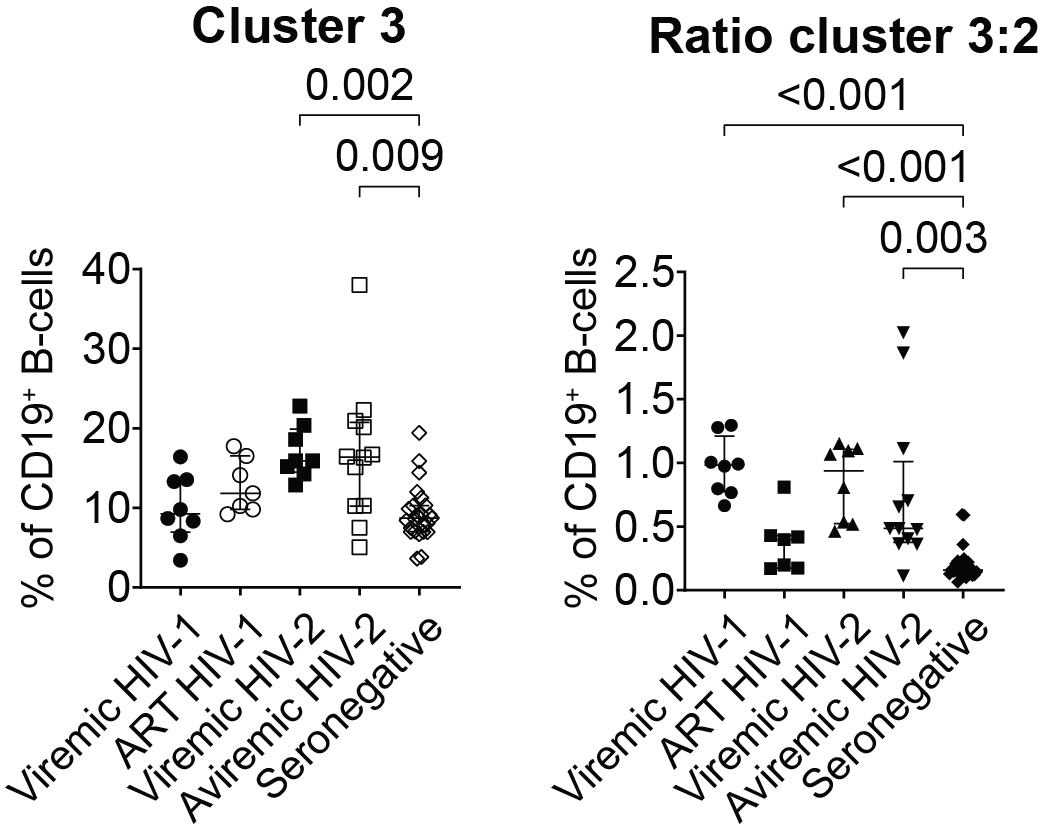

Supplement: Supplementary file 1 [file cells-11-03142-s001.zip › cells-1922271-supplementary/Figure S4.png]

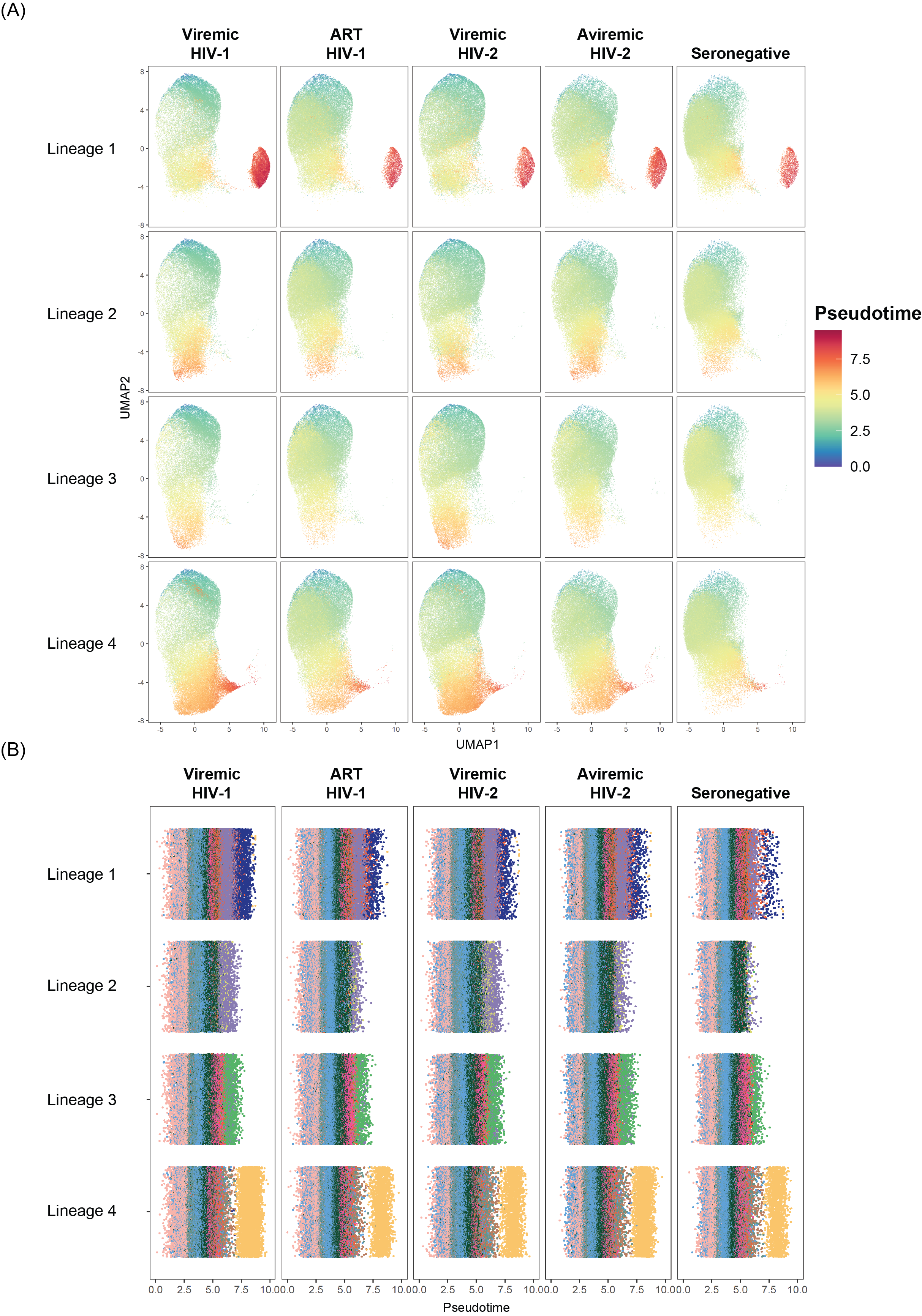

Supplement: Supplementary file 1 [file cells-11-03142-s001.zip › cells-1922271-supplementary/Figure S5.png]
